# Body Composition in Preschool Children and the Association With Prepregnancy Weight and Gestational Weight Gain: An Ambispective Cohort Study

**DOI:** 10.3389/fnut.2022.881452

**Published:** 2022-05-24

**Authors:** Fangfang Chen, Jing Wang, Zijun Liao, Xinnan Zong, Ting Zhang, Xianghui Xie, Gongshu Liu

**Affiliations:** ^1^Capital Institute of Pediatrics, Beijing, China; ^2^Tianjin Women’s and Children’s Health Center, Tianjin, China

**Keywords:** body composition, body fat, obesity, cohort study, gestational weight gain, prepregnant BMI, preschool children child adiposity, gestational weight

## Abstract

**Objective:**

To describe the body composition in preschool children and to evaluate the association with prepregnancy BMI and gestational weight gain (GWG).

**Methods:**

Children were recruited in their first year in kindergarten (3 years old) and followed up for the next 2 years. Information during pregnancy and birth was retrieved from medical records. Height, weight, fat mass, fat-free mass, and percentage of body fat (FM%) were measured through a bioelectrical impedance analysis for each child visit, and BMI, fat mass index (FMI), and fat-free mass index (FFMI) were calculated. Generalized linear mixed models (GLMMs) were used to evaluate the associations between prepregnancy weight, GWG, and adiposity indicators.

**Results:**

A total of 3,329 single-birth 3-year-old children were recruited as the baseline population and were followed at 4 and 5 years old. During the 3 years of follow-up, the mean (±SD) values of BMI, FMI, FFMI, and FM% of the children were 15.6 (±1.6) kg/m^2^, 2.8 (±1.3) kg/m^2^, 12.8 (±0.7) kg/m^2^, and 17.2% (±5.8%), respectively. The prevalence rates of overweight and obesity in mothers before pregnancy were 16.6 and 3.2%, respectively. Mothers were divided into three groups based on GWG: appropriate (1,233, 37.0%), excessive (767, 23.0%), and insufficient (1,329, 39.9%). GLMMs analyses showed that the preschool children’s BMI, FMI, FFMI, and FM% were all significantly positively related to maternal prepregnancy BMI and GWG (all *P* < 0.001); the children of mothers who were overweight/obese before pregnancy were more likely to be overweight/obese, high FMI, high FFMI, and high FM% at preschool age (all *P* < 0.001); although maternal excessive GWG was not correlated with offspring’s overweight/obese (*P* = 0.156), the children of mothers with excessive GWG are more likely to have higher FMI, but not to be with a higher FFMI status than the children of mothers with appropriate GWG. For prepregnancy overweight/obese women, compared with the GWG-appropriate group, maternal excessive GWG was related to the risk of high FMI (coefficient = 0.388, 95% CI: 0.129–0.647) and high FM% (coefficient = 0.352, 95% CI: 0.097–0.607), but was not related to the risk of overweight/obese or high FFMI of the offspring at preschool age.

**Conclusion:**

Fat mass index decreased with age, while FFMI increased with age among 3- to 5-year-old children. It is necessary to optimize maternal weight prior to conception and GWG management to improve the health outcomes of the offspring.

## Introduction

The worldwide prevalence of overweight and obesity has approximately doubled since 1980 to the extent that over one-third of the world’s population is now classified as overweight or obese (1). According to a prediction, 57.8% of the world population will be overweight or obese by 2030 if current trends continue (2). The prevalence of overweight/obesity in Chinese adults has increased linearly (3). The course of obesity can begin very early, especially *in utero* and in the first 2 years of life (4, 5). The prevalence of obesity among Chinese preschool children increased continuously from 1986 to 2016, especially after 3 years of age (6).

Body mass index (BMI) is a widely used indicator to evaluate general adiposity, but its use is limited because it does not discriminate for body composition (7). Parental environmental factors affect the health and chronic disease risk of people throughout their lives (8–10). An increased prevalence of prepregnancy obesity and excessive gestational weight gain (GWG) is associated with a child’s weight status or other components of metabolic disease both in childhood and adulthood, and it is time to redirect efforts and optimize maternal weight prior to conception (11–13).

Previous studies that evaluated the consequences of prepregnancy BMI or GWG for the child mostly focused on birth outcomes or the health outcomes at a single age point, and few studies followed up on the health conditions of offsprings at multiple time points, especially lack comprehensive assessment of the children’s growth and development, including not only anthropometric indicators but also multi-time evaluation of body composition. Studies with larger sample sizes and an extended follow-up period are also warranted to generate stronger evidence and to evaluate the influence of prepregnancy weight status and GWG on the long-term body composition of children.

This study prospectively followed preschool children in kindergartens for 3 years between the ages of 3 and 5 and linked their mothers’ medical records during pregnancy. This ambispective cohort study aimed to describe the body composition of preschool children and to evaluate whether and how prepregnancy weight status and GWG affect the body composition of 3- to 5-year-old children.

## Materials and Methods

This study was carried out by the Tianjin Women’s and Children’s Health Center and Capital Institute of Pediatrics in Tianjin from 2017 to 2020 and was approved by the IRB of Tianjin Women’s and Children’s Health Center (BGI-IRB 17116-201711). Parental written consent forms were obtained when the children were recruited in junior class.

### Participants

In general, children enter kindergarten at 3 years old in China, and they usually stay there for 3 years (junior, middle, and senior class) before school. Children were recruited at kindergarten junior classes with informed consent obtained from their parents, and their mothers’ information during pregnancy was matched by retrieval from medical records. The exclusion criteria for the children were as follows: (1) any condition or chronic diseases or use of any drug known to affect growth and development; (2) acute diseases that prohibit children from participating in the physical examination; (3) twins or other multiple births; (4) mothers without GWG records in the antenatal healthcare system.

By using a stratified cluster sampling method, 11 districts (including 6 central urban districts, 4 loop urban districts, and 1 suburban district) were selected from the 16 municipal districts in Tianjin, the city where the prevalence of obesity in school-aged children is the highest in China. Then, 42 state-owned kindergartens were selected from 11 districts starting in 2017. Children in the junior classes of the 42 kindergartens were recruited into the cohort study, which has good population representativeness of the preschool children in Tianjin, and they were followed up and completed physical examinations in junior, middle, and senior classes from September 2017 to September 2020. Their mothers’ clinical information on antenatal and postnatal healthcare was collected from the Tianjin Hospital healthcare system, including anthropometry results collected at each follow-up phase.

### Measurements of Gestational Weight

Prepregnancy weight and height were self-reported and registered at the first visit during the 1st trimester. Maternal gestational weight was measured at antenatal clinics during each visit. The median number of repeat weight measurements per woman was 7, and P_25_–P_75_ was 6–8 times. GWG was calculated as weight at the last visit (within 1 week of delivery) minus the self-reported weight for the first visit at the antenatal clinic.

### Anthropometric Measurements of Children

For each child followed up in kindergarten, height was measured without shoes at each visit. Bodyweight, fat mass, fat-free mass, and percentage of body fat mass (FM%) were measured by trained nutritionists through bioelectrical impedance analysis (see higher BAS-H, China), which measured the impedances of the body and each limb in the standing position and is suitable for children aged 3 years and older, at frequencies of 1 kHz, 5 kHz, 50 kHz, 250 kHz, 500 kHz, and 1 MHz. Children were required to be on fasting and to have an empty bladder. When in the measurement, children in light clothing stood on the platform without shoes, and held both hands at a 45-degree angle away from the body; four tactile electrodes were in contact with the palm and thumb of both hands, and the other four were in contact with the anterior and posterior aspects of the sole of both feet. BMI, fat mass index (FMI), and fat-free mass index (FFMI) were calculated for each subject as body weight, fat mass, and fat-free mass in kilograms divided by height in meters squared, respectively.

### Classification of Health Outcomes and Conditions

GWG was classified as insufficient, appropriate, or excessive according to the 2009 Institute of Medicine (IOM) criteria (14). Prepregnancy weight status was defined as two groups (BMI < 25 kg/m^2^ group and ≥ 25 kg/m^2^ overweight/obese group).

The Chinese gender-specific and age-specific BMI cutoffs for overweight and obesity for children from 2 to 18 years (15) were used to define preschool children as having normal weight, overweight, or obesity. The BMI cutoffs were established based on representative data from two national representative cross-sectional surveys in China: The National Growth Survey of Children under 7 years of age in the nine cities of China in 2005 and The Physical Fitness and Health Surveillance of Chinese School Students in 2005, with estimates of L, M, and S parameters, values of percentile and Z-score calculated. High FMI was defined as an age- and gender-specific Z-score of FMI at each follow-up ≥1 during the 3 years; low FFMI was defined as an age- and gender-specific Z-score of FFMI at each follow-up <–1 during the 3 years; and high FM% was defined as an age- and gender-specific Z-score of FM% at each follow-up ≥1 during the 3 years.

### Statistical Analyses

Data analyses were performed using SPSS 20.0 (SPSS, Inc., Chicago, Illinois) and R software (version 4.1.2). Comparisons of characteristics were conducted using one-way ANOVA or chi-square tests. The loess smooth curve of body composition indicators (95% CIs) with age during the 3-year follow-up was developed. Generalized linear mixed models (GLMMs) (16, 17) were used to evaluate the associations between prepregnancy weight, GWG, and the adiposity indicators, with a linear regression link or a binary logistic regression. Considering potential confounding factors (12), gestational week, parity, children’s age, and gender, whether exclusive breastfeeding within 6 months of birth, annual family income, maternal educational level, paternal educational level, maternal occupation, and paternal occupation were adjusted in the GLMMs.

## Results

### Participants and Cohort Follow-Up

**FIGURE 1:**
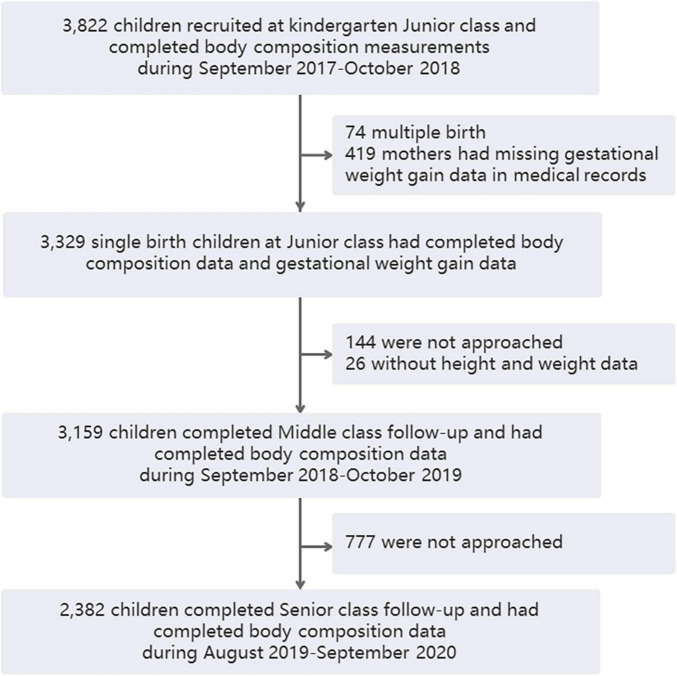
Flowchart of the study cohort.

A total of 3,822 children were recruited in their kindergarten junior class and completed physical examinations, including body composition analysis, from October 2017 to October 2018. Excluding 74 twins or other multiple-birth children and 419 mothers who had missing GWG data in medical records, 3,329 single-birth children had complete maternal GWG data in the antenatal healthcare system, and they provided the cohort baseline data. In total, 3,329 children remained participants and the other 419 children were non-participants. There were no significant differences in demographic and growth parameters between the participant and non-participant groups in the kindergarten junior class except that the FFMI showed a slight difference (12.73 vs. 12.86 kg/m^2^, Table 1). When the children were followed up through kindergarten middle class, 144 of them were not approached, 26 of them did not have height and weight examinations, and 3,159 remained in the study. In the senior kindergarten class, 2,382 children were followed up and had complete adiposity indicator data (Figure 1). A total of 144 and 777 children were not approached during the middle and senior years because they did not continue staying in the kindergartens.

**TABLE 1:** Characteristics of children at junior class with and without GWG data that as participants and non-participants in the follow-up study.

	Participants (*n* = 3,329)	Non-participants (*n* = 419)	*P*-value
Age, mean (SD), y	3.79 (0.3)	3.78 (0.31)	0.450
Male, n (%)	1,732 (0.52)	282 (0.53)	0.737
Weight, mean (SD), kg	16.53 (2.31)	16.55 (2.58)	0.837
Height, mean (SD), cm	102.77 (4.51)	102.46 (4.43)	0.186
BMI, mean (SD), kg/m^2^	15.6 (1.43)	15.7 (1.53)	0.185
FMI, mean (SD), kg/m^2^	2.87 (1.15)	2.84 (1.2)	0.623
FFMI, mean (SD), kg/m^2^	12.73 (0.73)	12.86 (0.76)	0.001
Overweight, n (%)	408 (0.13)	49 (0.13)	0.803
Obese, n (%)	177 (0.06)	27 (0.07)	0.353

*BMI, body mass index; FMI, fat mass index; FFMI, fat free mass index.*

*Overweight and obesity was defined according to the Chinese gender-specific and age-specific BMI cutoffs for overweight and obesity for children from 2 to 18 years of age.*

### Characteristics of the Participants

**FIGURE 2:**
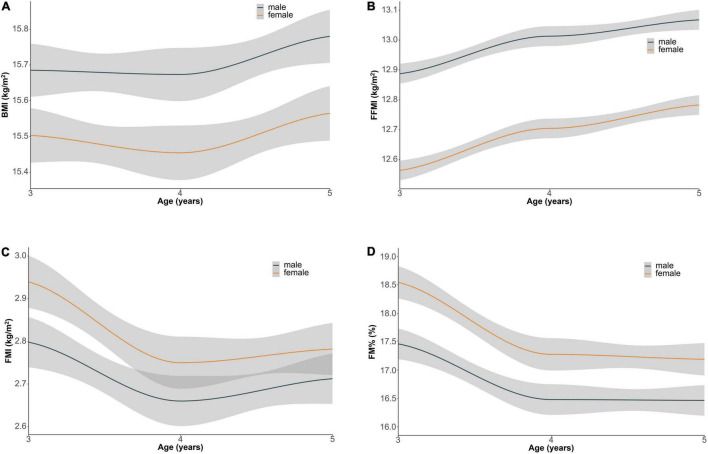
Loess smooth curve of body composition indicators (95% CIs) with age during 3-year follow-up. **(A)** BMI, **(B)** FFMI, **(C)** FMI, and **(D)** FM%. BMI, body mass index; FFMI, fat free mass index; FMI, fat mass index; FM%, percentage of body fat mass.

During the 3 years of follow-up, the mean (±SD) values of BMI, FMI, FFMI, and FM% of the children were 15.6 (±1.6) kg/m^2^, 2.8 (±1.3) kg/m^2^, 12.8 (±0.7) kg/m^2^, and 17.2% (± 5.8%), respectively. The change trends of adiposity indicators (95% CIs) with age during the 3-year follow-up are shown in Figure 2. FMI decreased with age, while FFMI increased with age among 3- to 5-year-old children.

As shown in Table 2, mothers were divided into three groups based on the 2009 IOM guidelines of GWG: appropriate (37.0%), excessive (23.0%), and insufficient (39.9%). Child gender and age during the 3-year follow-up were not significantly different between the prepregnancy BMI <25 kg/m^2^ and ≥25 kg/m^2^ groups. Parity, child gender, gestational week, and age at kindergarten during the 3-year follow-up were not significantly different among the three different GWG groups.

**TABLE 2:** Characteristics of the mothers and their preschool offspring in different groups (*n* = 3,329).

	Prepregnancy BMI		*P*-value*	Gestational weight gain			*P*-value*
	<25 kg/m^2^ (*n* = 2,670)	≥25 kg/m^2^ (*n* = 659)		Insufficient (*n* = 1,329)	Appropriate (*n* = 1,233)	Excessive (*n* = 767)	
	Mean (SD)/n (%)	Mean (SD)/n (%)		Mean (SD)/n (%)	Mean (SD)/n (%)	Mean (SD)/n (%)	
**Mother characteristics**							
Pre-pregnancy BMI (kg/m^2^)	21 (2.1)	27.7 (2.4)	<0.001	21.5 (2.9)	22.4 (5.3)	24.2 (9.1)	<0.001
Gestational weight gain (kg)	12.2 (4.0)	10.7 (4.4)	<0.001	8.4 (2.3)	12.6 (2.3)	16.7 (3.4)	<0.001
**Parity**							
1	2,345 (81.6)	530 (18.4)	<0.001	1,130 (39.3)	1,071 (37.3)	674 (23.4)	0.130
2	317 (71.9)	124 (28.1)		196 (44.4)	157 (35.6)	88 (20.0)	
3	8 (61.5)	5 (38.5)		3 (23.1)	5 (38.5)	5 (38.5)	
Total	2,670 (80.2)	659 (19.8)		1,329 (39.9)	1,233 (37.0)	767 (23.0)	
**Children characteristics**							
**Gender**							
Male	1,412 (81.5)	320 (18.5)	0.047	688 (51.8)	660 (53.5)	384 (50.1)	0.312
Female	1,258 (78.8)	339 (21.2)		641 (48.2)	573 (46.5)	383 (50.0)	
Total	2,670 (80.2)	659 (19.8)		1,329 (39.9)	1,233 (37.0)	767 (23.0)	
Gestational weeks (week)	39 (1.3)	38.9 (1.5)	0.003	39.0 (1.5)	39.0 (1.3)	39.0 (1.4)	0.694
**Age 3-at Kindergarten Junior class**							
Age (years)	3.8 (0.3)	3.8 (0.3)	0.608	3.8 (0.33)	3.8 (0.3)	3.8 (0.3)	0.973
FM% (%)	17.6 (5.4)	19.6 (6.0)	<0.001	17.3 (5.4)	18.2 (5.5)	18.9 (5.9)	<0.001
FMI (kg/m^2^)	2.8 (1.1)	3.2 (1.3)	<0.001	2.7 (1.05)	2.9 (1.5)	3.1 (1.3)	<0.001
FFMI (kg/m^2^)	12.7 (0.7)	12.9 (0.8)	<0.001	12.7 (0.73)	12.8 (0.7)	12.8 (0.8)	<0.001
BMI (kg/m^2^)	1.6 (15.5)	1.3 (16.2)	<0.001	15.4 (1.3)	15.7 (1.5)	15.9 (1.6)	<0.001
Overweight	284 (10.6)	124 (18.8)	<0.001	123 (9.3)	170 (13.8)	115 (15.0)	<0.001
Obese	103 (3.9)	74 (11.2)		45 (3.4)	71 (5.8)	61 (8.0)	<0.001
**Age 4-at Kindergarten-Middle class**							
Age (years)	4.6 (0.3)	4.6 (0.3)	0.821	4.6 (0.3)	4.6 (0.3)	4.6 (0.3)	0.376
FM% (%)	16.4 (5.6)	18.8 (6.8)	<0.001	16.1 (5.6)	17 (6.0)	17.9 (6.2)	<0.001
FMI (kg/m^2^)	2.6 (1.2)	3.2 (1.5)	<0.001	2.5 (1.2)	2.7 (1.3)	2.9 (1.4)	<0.001
FFMI (kg/m^2^)	12.8 (0.7)	13.1 (0.7)	<0.001	12.8 (0.7)	12.9 (0.7)	13.0 (0.8)	<0.001
BMI (kg/m^2^)	15.4 (1.5)	16.2 (1.9)	<0.001	15.3 (1.5)	15.6 (1.7)	15.9 (1.7)	<0.001
Overweight	267 (10.6)	121 (19.2)	<0.001	124 (9.9)	146 (12.6)	118 (15.9)	<0.001
Obese	141 (5.6)	98 (15.6)		58 (4.6)	92 (7.9)	89 (12.0)	<0.001
**Age 5-at Kindergarten-Senior class**							
Age (years)	5.6 (0.3)	5.6 (0.3)	0.181	5.6 (0.3)	5.6 (0.3)	5.6 (0.3)	0.832
FM% (%)	15.9 (6.3)	19.6 (8.5)	<0.001	15.8 (6.5)	16.7 (6.9)	18.1 (7.7)	<0.001
FMI (kg/m^2^)	2.6 (1.4)	3.5 (2.1)	<0.001	2.5 (1.4)	2.8 (1.5)	3.1 (1.9)	<0.001
FFMI (kg/m^2^)	12.9 (0.8)	13.2 (0.9)	<0.001	12.9 (0.8)	13.0 (0.8)	13.1 (0.9)	<0.001
BMI (kg/m^2^)	15.5 (1.8)	16.7 (2.5)	<0.001	15.4 (1.8)	15.7 (2.0)	16.2 (2.3)	<0.001
Overweight	187 (11.0)	74 (17.9)	<0.001	83 (9.8)	106 (13.6)	72 (14.7)	<0.001
Obese	117 (6.9)	84 (20.3)	<0.001	51 (6.0)	77 (9.9)	73 (14.9)	<0.001

*One-way ANOVA or chi-square test.

*BMI, body mass index; FM%, percentage of body fat mass; FMI, fat mass index; FFMI, fat free mass index.*

For maternal characteristics, the mean prepregnancy BMI was 22.5 (*SD* = 5.8) kg/m^2^, and the prevalence rates of overweight and obesity of mothers before pregnancy were 16.6 and 3.2%, respectively.

Of the children during the 3-year follow-up in kindergarten, BMI, FMI, FFMI, and FM% were all significantly different between the prepregnancy BMI <25 kg/m^2^ and ≥25 kg/m^2^ groups, and these indicators were also significantly different among the three GWG groups (all *P* < 0.001). The chi-squared test showed that the prevalence of overweight and obesity during the three years were all significantly different between the prepregnancy BMI <25 kg/m^2^ and ≥25 kg/m^2^ groups (all *P* < 0.001), and the chi-squared test showed that these indicators increased with increasing GWG status (all *P*_*for trend*_ < 0.001).

### Associations Between Prepregnancy BMI, Gestational Weight Gain, and Adiposity Indicators in Kindergarten Children

Adjusted by gestational week, parity, age and gender of children, annual family income, exclusive breastfeeding within 6 months of birth, maternal educational level, paternal educational level, maternal occupation, and paternal occupation, GLMMs analyses with a linear regression link between target distribution and relationship showed that the adiposity indicators of the offspring were all significantly positively related to maternal prepregnancy BMI and GWG (all *P* < 0.001) (Table 3).

**TABLE 3:** Generalized Linear Mixed Models (GLMMs) associations between prepregnancy BMI, GWG and the adiposity indicators in kindergarten children.

Dependent variable	Main effect	Estimate	95%*CI*		S.E.	*t*-value	*P*-value	*F* value of the GLMM models	*P*-value of the GLMM models
			Lower	Upper					
BMI	Prepregnancy BMI	0.139	0.129	0.148	0.005	28.754	<0.001	22.030	<0.001
	GWG	0.042	0.035	0.050	0.004	10.557	<0.001		
FMI	Prepregnancy BMI	0.094	0.086	0.102	0.004	24.047	<0.001	15.918	<0.001
	GWG	0.027	0.021	0.033	0.003	8.279	<0.001		
FFMI	Prepregnancy BMI	0.046	0.042	0.050	0.002	20.670	<0.001	16.273	<0.001
	GWG	0.016	0.012	0.020	0.002	8.557	<0.001		
FM%	Prepregnancy BMI	0.407	0.371	0.443	0.018	22.065	<0.001	14.814	<0.001
	GWG	0.113	0.082	0.143	0.015	7.331	<0.001		

*Prepregnancy BMI, GWG, gestational week, parity, children’s age and gender, annual family income, whether exclusive breastfeeding within 6 months of birth, maternal educational level, paternal educational level, maternal occupation, and paternal occupation were entered in the GLMMs, adiposity indicators during the 3 years’ follow were dependent variables.*

*BMI, body mass index; FMI, fat mass index; FFMI, fat free mass index; FM%, percentage of fat mass.*

### Associations Between Prepregnancy Weight Status, Gestational Weight Gain Groups, and Adiposity Classification Indicators

After the diagnosis of the adiposity indicators, GLMMs analyses with a binary logistic regression link between target distribution and relationship were executed. Compared to the children of mothers with appropriate GWG, insufficient GWG was a protective factor against overweight/obesity, high FMI, and high FM%, but they were more likely to have a low FFMI at preschool age (all *P* < 0.01). Although excessive maternal GWG was not correlated with offsprings’ overweight/obese (*P* = 0.156), the children of mothers with excessive GWG were more likely to have a higher FMI (coefficient = 0.179, 95% CI: 0.015–0.342) but not correlated with the possibility of having a higher FFMI (P = 0.064) compared with the children of mothers with appropriate GWG. Compared with the children of prepregnancy BMI <25 kg/m^2^ mothers, the children of mothers who were overweight/obese before pregnancy were more likely to be overweight/obese (coefficient = 0.779, 95% CI: 0.647–0.912), and have a high FMI/high FFMI/high FM% at preschool age (coefficient: 0.755, 0.563, 0.658, respectively, and all *P* < 0.001) (Table 4).

**TABLE 4:** Generalized Linear Mixed Models (GLMMs) associations between prepregnancy weight status, GWG groups and the adiposity indicators in kindergarten children.

Dependent variable	Main effect	*F* value of the GLMM models	*P*-value of the GLMM models	Estimate	95%*CI*		S.E.	*t*-value	*P*-value
					Lower	Upper			
Overweight/obese	Prepregnancy overweight/obese*	132.880	<0.001	0.779	0.647	0.912	0.068	11.527	<0.001
	GWG insufficient^#^	17.699	<0.001	–0.340	–0.480	–0.200	0.071	–4.764	<0.001
	GWG excessive^#^			0.103	–0.039	0.245	0.073	1.418	0.156
High FMI	Prepregnancy overweight/obese*	96.667	<0.001	0.755	0.604	0.905	0.077	9.832	<0.001
	GWG insufficient^#^	10.728	<0.001	–0.247	–0.413	–0.080	0.085	–2.906	0.004
	GWG excessive^#^			0.179	0.015	0.342	0.083	2.140	0.032
High FFMI	Prepregnancy overweight/obese*	57.276	<0.001	0.563	0.417	0.709	0.074	7.568	<0.001
	GWG insufficient^#^	9.103	<0.001	–0.217	–0.369	–0.065	0.078	–2.794	0.005
	GWG excessive^#^			0.148	–0.009	0.304	0.080	1.851	0.064
Low FFMI	Prepregnancy overweight/obese*	20.171	<0.001	–0.421	–0.605	–0.237	0.094	–4.491	<0.001
	GWG insufficient^#^	4.693	0.009	0.232	0.082	0.383	0.077	3.029	0.002
	GWG excessive^#^			0.081	–0.098	0.261	0.092	0.887	0.375
High FM%	Prepregnancy overweight/obese*	78.523	<0.001	0.658	0.512	0.804	0.074	8.861	<0.001
	GWG insufficient^#^	9.410	<0.001	–0.252	–0.408	–0.096	0.080	–3.172	0.002
	GWG excessive^#^			0.117	–0.040	0.274	0.080	1.463	0.144

*Prepregnancy BMI, GWG, gestational week, parity, children’s age and gender, annual family income, whether exclusive breastfeeding within 6 months of birth, maternal educational level, paternal educational level, maternal occupation and paternal occupation were entered in the GLMMs, adiposity indicators during the 3 years’ follow were dependent variables.*

*Prepregnancy normal weight group as reference.

*^#^Appropriate gestational weight gain group as reference. BMI, body mass index; FMI, fat mass index; FFMI, fat free mass index; FM%, percentage of fat mass.*

Stratified by prepregnancy weight status (BMI < 25 kg/m^2^ and ≥ 25 kg/m^2^), associations between GWG groups and the adiposity indicators are shown in Table 5. For prepregnancy BMI <25 kg/m^2^ women, compared to the GWG-appropriate group, maternal excessive GWG was not related to the risk of overweight/obesity, high FMI, or high FM% of the offspring but was related to high FFMI (coefficient = 0.248, 95% CI: 0.048–0.447). GWG insufficiency was a protective factor against overweight/obesity, high FMI, and high FM%, but it was a risk factor for low FFMI (coefficient = 0.238, 95% CI: 0.095–0.382). For prepregnancy overweight/obese women, compared with the GWG-appropriate group, maternal excessive GWG was not related to overweight/obese or high FFMI of the offspring at preschool age but was related to the risk of high FMI (coefficient = 0.388, 95% CI: 0.129–0.647) and high FM% (coefficient = 0.352, 95% CI: 0.097–0.607), which provides supporting evidence for the association between maternal weight and offsprings’ body composition at preschool age.

**TABLE 5:** Generalized Linear Mixed Models (GLMMs) associations between GWG groups and the adiposity indicators in kindergarten children, stratified by prepregnancy weight status.

Dependent variable	Main effect^#^	*F*-value of the GLMM models	*P*-value of the GLMM models	Estimate	S.E.	*t*-value	*P*-value	95%*CI*	
								Lower	Upper
**Prepregnancy BMI < 25 kg/m^2^**									
Overweight/obese	GWG insufficient	14.991	<0.001	–0.348	0.0757	–4.602	<0.001	–0.497	–0.200
	GWG excessive			0.080	0.0928	0.864	0.388	–0.102	0.262
High FMI	GWG insufficient	5.016	0.007	–0.259	0.0896	–2.888	0.004	–0.435	–0.083
	GWG excessive			0.004	0.1117	0.031	0.975	–0.216	0.223
High FFMI	GWG insufficient	6.753	0.001	–0.119	0.0829	–1.442	0.149	–0.282	0.043
	GWG excessive			0.248	0.1019	2.430	0.015	0.048	0.447
Low FFMI	GWG insufficient	6.001	0.002	0.238	0.0731	3.262	0.001	0.095	0.382
	GWG excessive			0.036	0.0994	0.361	0.718	–0.159	0.231
High FM%	GWG insufficient	5.482	0.004	–0.264	0.0828	–3.190	0.001	–0.427	–0.102
	GWG excessive			–0.047	0.1051	–0.450	0.653	–0.253	0.159
**Prepregnancy BMI ≥ 25 kg/m^2^**									
Overweight/obese	GWG insufficient	5.341	0.005	–0.391	0.1827	–2.141	0.032	–0.750	–0.033
	GWG excessive			0.187	0.1206	1.552	0.121	–0.049	0.424
High FMI	GWG insufficient	8.391	<0.001	–0.325	0.2091	–1.554	0.120	–0.735	0.085
	GWG excessive			0.388	0.1319	2.939	0.003	0.129	0.647
High FFMI	GWG insufficient	3.012	0.049	–0.434	0.2047	–2.122	0.034	–0.836	–0.033
	GWG excessive			0.056	0.1332	0.424	0.672	–0.205	0.318
Low FFMI	GWG insufficient	Model analysis is not feasible							
	GWG excessive								
High FM%	GWG insufficient	6.746	0.001	–0.262	0.2034	–1.289	0.198	–0.661	0.137
	GWG excessive			0.352	0.1300	2.704	0.007	0.097	0.607

*Prepregnancy BMI, GWG, gestational week, parity, children’s age and gender, annual family income, whether exclusive breastfeeding within 6 months of birth, maternal educational level, paternal educational level, maternal occupation, and paternal occupation were entered in the GLMMs, adiposity indicators during the 3 years’ follow were dependent variables.*

*^#^Appropriate gestational weight gain group as reference.*

*BMI, body mass index; FMI, fat mass index; FFMI, fat free mass index; FM%, percentage of fat mass.*

## Discussion

A meta-analysis (18) demonstrated that 27.8% of women had insufficient GWG and 39.4% had excessive GWG in the global population compared with the meta-analysis results in an Asian population. The total prevalence of insufficient GWG in our study was similar to that of an Asian population (39.9 vs. 35.6%), and the rate of excessive GWG in our sample was higher than that in the Asian population (23.0 vs. 16.8%).

A systematic review showed that prepregnancy maternal overweight is associated with higher offspring adiposity, and the relationship between maternal gestational weight gain and offspring lean mass or fat-free mass was not consistent (12). Based on the 3-year follow-up of the offspring, the present cohort study illustrated that both prepregnancy status and maternal GWG were related to preschool child adiposity. Overall, BMI, FMI, FFMI, and FM% in preschool children increased with increasing prepregnancy BMI and GWG. Preconception care offers a unique opportunity to address the pressing public health goal of reducing pregnancy-related morbidity and mortality (19). Women who plan to become pregnant should, before becoming pregnant, receive care that includes lifestyle changes, such as smoking cessation, guidance on exercise habits, dietary intake, and the use of vitamins and supplements. Preconception assessment of nutritional status should identify those individuals who are underweight or overweight (20). Our results also indicated that women with overweight/obesity before pregnancy should pay more attention in maintaining appropriate weight gain during pregnancy. Because excessive maternal GWG increased the risks of high FMI in offspring at preschool age, but is not related to classified high FFMI status, especially among prepregnancy overweight/obese mothers. This study demonstrated that maternal weight not only has an impact on the birth outcome but also influences the body composition of the offspring at preschool age. The results provided supporting evidence of the long-term impact of maternal weight and presented core component data for guiding clinical practice, including preconception care and prenatal care.

Excessive GWG is a risk factor for cesarean delivery, large for gestational age (LGA), and macrosomia (21). GWG has significant health implications for both the mother and the child, and it is one of the non-genetic-based associations between maternal and infantile excess body mass; this demonstrates an independent role in mother-to-child obesity transmission (22). Considering that this study has good population representativeness of preschool children in Tianjin, where the prevalence of obesity in school-aged children is the highest in China (23), more attention should be given, and increasingly effective interventions should be implemented on weight gain control during pregnancy in obstetrics clinics in Tianjin, especially among women who are overweight and obese before pregnancy, to control childhood obesity and diminish mother-to-child obesity transmission.

Children with obesity before puberty can develop obesity in early adulthood, with early-life fat deposition associated with a later risk of adult obesity (24, 25). The WHO developed guidelines on physical activity, sedentary behavior, and sleep for children under 5 years of age in 2016 (26) and 2019 (27), which called for more political commitment to face the increasing problem of childhood obesity with specific practical implementations for children. BMI has been widely used as a simple measure of defining obesity. However, it cannot differentiate between fat mass and fat-free mass (7, 28). Furthermore, simply relying on BMI to assess obesity could hinder future interventions aimed at obesity prevention and control (1). Prepregnancy BMI and GWG have attracted significant scientific attention, yet little is known about maternal factors that influence children’s health in the long term (22). In this prospective cohort of preschool children from kindergarten in our study, body composition was measured in all 3 years. Combined with our previous results, the fat-free mass had a protective impact on impaired fasting glucose in children with normal weight but not in children with overweight and obesity (29). This calls for urgent attention to the importance of maintaining normal weight before pregnancy and avoiding excessive GWG because the children of mothers with excessive GWG may have higher FMI, which is harmful to their health, and not more muscle or bone mass.

The strength of this study is that both the continuous follow-up of child adiposity development in the 3 years of kindergarten and obstetric records during pregnancy were included in an ambispective cohort study. Some potential confounding variables were adjusted in the analysis which reveals the relatively causal link between prepregnancy BMI, GWG, and preschool child adiposity at the body composition level. Body composition and anthropometric measurements were involved in the continuous 3-year measurements, which made it possible to draw continuous lines during the 3 years in preschool children. Furthermore, maternal gestational weight was measured at antenatal clinics at each visit, and the medical records of the gestational weight improved the reliability of the GWG data.

Our study has some limitations. First, prepregnancy weight was self-reported at the first visit to the antenatal clinic: there may be a recall bias in the self-reported weight data. Although a systematic review concluded that self-reported pregnancy-related weight has a high correlation with weight measurement (30), women generally underestimate their own weights (31), which may lead to a higher GWG than the actual value. Furthermore, the association between GWG and preschool child adiposity can be enhanced if this is the case. Another limitation is due to the ambispective cohort study method: we failed to retrieve some of the maternal GWG data records, and there was a different loss of follow-up rates among children in 3 years, which could have resulted in bias. Third, we measured the children’s adiposity indices using bioelectrical impedance analysis rather than the gold standard, which improves the feasibility, but the accuracy should be further considered. Fourth, lifestyle behaviors, such as maternal smoking, diet, sleep, and physical activities, might be confounding factors for the relationship between pregnancy weight and offsprings’ body composition, but they were not collected ideally, so they were not adjusted in the analysis. In addition, the percentage of mothers with prepregnancy BMI <18.5 kg/m^2^ was 10.6%. Limited by the sample size, prepregnancy BMI of 25 kg/m^2^ was used to divide the mothers into two groups, which may have a certain impact on the analysis results when combined with underweight and overweight as a reference category.

## Conclusion

Fat mass index decreased with age, while FFMI increased with age among 3- to 5-year-old children. Maternal overweight/obesity before pregnancy increases the risk of overweight/obesity, high FMI, high FFMI, and high FM% in preschool offspring. The preschool children of prepregnancy overweight/obese mothers with excessive GWG are more likely to have a higher FMI and FM% level but not a classified high FFMI level, which is required to remain healthy. It is necessary to optimize maternal weight prior to conception and GWG management to improve the health outcomes of the offspring.

## Data Availability Statement

The datasets presented in this article are not readily available because the fund supporting the project does not allow the research data to be transferred to others or other institutions without consent. Requests to access the datasets should be directed to FC, airechen@126.com.

## Ethics Statement

The studies involving human participants were reviewed and approved by the IRB of Tianjin Women’s and Children’s Health Center (BGI-IRB 17116-201711). Written informed consent to participate in this study was provided by the participants’ legal guardian/next of kin.

## Author Contributions

FC conceptualized and designed the study, carried out the analyses, drafted the manuscript, and reviewed and revised the manuscript. XX and GL conceptualized and designed the study, supervised data analyses, and reviewed the manuscript. TZ conceptualized and designed the study and supervised data analyses. JW, ZL, and XZ involved in data acquisition and critically reviewed and revised the manuscript. All authors critically reviewed the manuscript for interpretation and intellectual content and approved the final manuscript as submitted.

## Conflict of Interest

The authors declare that the research was conducted in the absence of any commercial or financial relationships that could be construed as a potential conflict of interest.

## Publisher’s Note

All claims expressed in this article are solely those of the authors and do not necessarily represent those of their affiliated organizations, or those of the publisher, the editors and the reviewers. Any product that may be evaluated in this article, or claim that may be made by its manufacturer, is not guaranteed or endorsed by the publisher.
